# Long-term Efficacy and Safety of Tacrolimus Plus Ustekinumab Combination Therapy in Children With Steroid-refractory Ulcerative Colitis

**DOI:** 10.1093/ibd/izae013

**Published:** 2024-01-24

**Authors:** Ryusuke Nambu, Ayako Miyazawa, Masashi Yoshida, Tomoko Hara, Itaru Iwama

**Affiliations:** Division of Gastroenterology and Hepatology, Saitama Children’s Medical Center, Saitama, Japan; Division of Gastroenterology and Hepatology, Saitama Children’s Medical Center, Saitama, Japan; Division of Gastroenterology and Hepatology, Saitama Children’s Medical Center, Saitama, Japan; Division of Gastroenterology and Hepatology, Saitama Children’s Medical Center, Saitama, Japan; Division of Gastroenterology and Hepatology, Saitama Children’s Medical Center, Saitama, Japan

**Keywords:** steroid-refractory ulcerative colitis, pediatric UC, tacrolimus, ustekinumab

Ulcerative colitis (UC) is particularly likely to be acute and severe in children.^1^ When infliximab and calcineurin inhibitors (CNI) fail to induce remission or immunomodulators fail to maintain remission after the success of induction of remission with CNI in steroid-refractory UC, a subsequent strategy is challenging. In such cases, ustekinumab monotherapy had a remission rate of only 15% at 8 weeks.^2^ Further, ustekinumab appears to achieve efficacy slowly in severe cases.^3^ Here we report efficacy and side effects of tacrolimus plus ustekinumab (Tac/UST) in childhood-onset steroid-refractory UC.

Eight patients (3 female) were treated with Tac/UST (Table 1). Median age was 11.5 years at diagnosis and 14.5 years at Tac/UST initiation. All had pancolitis and prior tumore necrosis factor (TNF)-α inhibitor treatment. The TNF-α inhibitor treatment had resulted in primary failure in 7 patients and infusion reaction in 1. (Secondary failure occurred with golimumab.) Median pediatric ulcerative colitis activity index at Tac/UST initiation was 55. Combination therapy has been maintained for over 1 year in 7 patients and 10 months in 1 patient. The steroid-free clinical remission rate at 8 weeks was 75% (6 of 8) and 57% (4 of 7) at 52 weeks. In 3 of 7, mucosal healing was confirmed beyond 52 weeks. In all, Tac blood trough concentrations were initially managed at 10 to 15 ng/mL (minimum for 2 weeks, maximum for 4 weeks) and thereafter at 5 to 10 ng/mL. Median duration of Tac treatment in 8 patients was 4.5 months; 1 discontinued treatment at 1 month because of UC exacerbation, and another at 4 months due to limb numbness. No complications such as infection or creatinine elevation occurred.

**Table 1.** Characteristics and outcomes of patients undergoing Tac/UST therapy.

		Before Tac/UST			After Tac/UST				
		Age at Uc Diagnosis (Years)	Use of Biologics	Response To Anti-TNF-α	Age at Tac/Ust Initiation (Years)	Pucai at Tac/Ust Initiation	Duration of Tac Use (Months)	Outcome	Side Effects
1	Female	11	IFX, GLM	IFX: IR GLM: LOR	22	55	7	10 mo SFCR	None
2	Female	10	IFX, GLM	Primary failure	10	55	8	18 mo SFCR & MH	None
3	Female	12	IFX	Primary failure	12	55	3	24 mo SFCR & MH	None
4	Male	15	IFX, VDL	Primary failure	16	65	5	42 mo SFCR & MH	None
5	Female	9	ADA, IFX, VDL	Primary failure	14	40	5	Flare up 21 months after Tac/UST	None
6	Male	14	IFX, VDL	Primary failure	15	70	4	Flare up 6 months after Tac/UST	None
7	Female	14	ADA	Primary failure	15	40	4	Flare up 4 months after Tac/UST	Numbness of hands and feet
8	Male	10	IFX	Primary failure	11	85	1	Failure to induce remission	None

Abbreviations: Tac, tacrolimus; UST, ustekinumab; UC, ulcerative colitis; IFX, infliximab; GLM, golimumab; VDL, vedolizumab; ADA, adalimumab;.

IR, infusion reaction; LOR, loss of response; PUCAI, pediatric ulcerative colitis activity index; SFCR, steroid-free clinical remission; MH, mucosal healing.

Here we describe long-term results of Tac/UST in moderate to severe childhood-onset UC with steroid and TNF-α inhibitor failures. Gisbert et al suggested tofacitinib, cyclosporine plus vedolizumab, or cyclosporine plus UST after anti-TNF-α failure in acute severe UC.^4^ Danese et al advocated combination treatment with 2 biologic agents or 1 biologic plus a small-molecule drug for refractory IBD.^5^ Prior assessments of Tac/UST have been limited to adults with refractory UC in short-term studies.^6,7^ Combinations of CNI and vedolizumab have been reported in UC, but mostly for patients naïve to biologics.^8–10^ Tacrolimus plus ustekinumab should be considered as treatment for children with steroid-refractory UC and studied in randomized controlled trials.

## Data Availability

The raw data supporting the conclusions of this article will be made available by the authors, with no undue reservation.
